# Caudal septoplasty: efficacy of a surgical technique-preliminnary report

**DOI:** 10.1590/S1808-86942011000200007

**Published:** 2015-10-19

**Authors:** Leonardo Bomediano Sousa Garcia, Pedro Wey de Oliveira, Tatiana de Aguiar Vidigal, Vinicius de Magalhães Suguri, Rodrigo de Paula Santos, Luis Carlos Gregório

**Affiliations:** 1 Rhinology Fellow - UNIFESP-EPM, MSc student in Rhinology -UNIFESP-EPM; 2 Rhinology Fellow - UNIFESP-EPM, MSc student in Rhinology -UNIFESP-EPM; 3 Rhinology Fellow - UNIFESP-EPM, MSc student in Rhinology -UNIFESP-EPM; 4 MSc in Otolaryngology - UNIFESP-EPM, Coordinator of the Rhinoseptoplasty Ward - UNIFESP-EPM; 5 PhD in Otolaryngology - UNIFESP-EPM, Head of the Rhinology Department - UNIFESP-EPM; 6 Professor - PhD - UNIFESP-EPM, Head of the Otorhinolaryngology Program - UNIFESP-EPM. Universidade Federal de São Paulo - Escola Paulista de Medicina, UNIFESP-EPM

**Keywords:** nasal cartilages, questionnaires, rhinometry, acoustic, nasal septum, prospective studies

## Abstract

Although not being the most frequent nasal septal deviations, those of the caudal septum account for many complaints. The correction of such defects has always been the subject of much controversy, and several different operative techniques have been described.

**Aim:**

To assess the efficacy of a surgical technique for correcting caudal septal deviations.

**Materials and Methods:**

Prospective study with preliminary reports of 10 patients who answered a standardized, specific questionnaire (the Nasal Obstruction Symptom Evaluation, or NOSE), underwent acoustic rhinometry and had their noses photographed. Caudal deviations were then corrected through a surgical technique whereby the entire deviated portion is removed and a straight cartilage segment is placed between the medial crura of the alar cartilages, through a retrograde approach, to support the nasal tip. Sixty days after all patients were reassessed.

**Results:**

As for the NOSE questionnaire, mean pre-operative and post-operative scores were 82.39 and 7.39 respectively (*p*<0.001). Pre-operative acoustic rhinometry showed mean minimum crosssectional area (MCA) values of 0.352 and 0.431 cm2, whereas mean post-operative values were 0.657 and 0.711 cm2(*p*<0.0001).

**Conclusions:**

The study results prove, both subjectively (patient satisfaction as measured with a standardized questionnaire) and objectively (acoustic rhinometry findings), that the proposed technique for correction of caudal septal deviation is safe and effective.

## INTRODUCTION

Caudal or anterior nasal septum deviations, despite not being the most common type, cause much complaint, both obstructive as well as cosmetic to the nasal tip. Guyuron et al. showed in a series of patients that only 5% of these patients had caudal deviations, Sedwick et al. found deviations in this area in 8% of 2,043 cases assessed^1^, ^2^.

Even small anterior deviations cause important nasal obstruction because they are located exactly in the narrowest portion of the nasal cavity, the nasal valve. Studies carried out by Grymer et al. used pre and post-operative acoustic rhinometry measures to prove that the nasal obstruction impact caused by minimum anterior nasal septum deviations is much greater than the one caused by large posterior deviations^3^. Patients with this type of deviation were the ones who benefited the most from the surgical correction according to Dinis et al.,^4^ in their analysis of long term patient satisfaction after septoplasty.

Besides the important functional problems, anterior septum deviations also cause clear cosmetic defects. These change the relation between the columella and the nostrils, causing significant defects on nasal tip position and symmetry^2^.

Thus, numerous techniques have been used to correct nasal septum caudal deviations. Since Metzembaum presented his caudal septoplasty technique in 1929, known as “swinging door”^5^, many other authors have developed different ways to correct these deviations. Nonetheless, having so many different techniques which have been tested and proved, reflect the great difficulty in correcting these anterior deviations. Should this be a simple correction, there would be only one single universal technique accepted.

The techniques which have been traditionally described to correct caudal nasal septum deviations only remove more posterior portions of the cartilage, sparing the anterior deviated portion, doing more conservative procedures such as mobilizations, sutures or weakening this portion. Thus, although not causing cosmetic harm to the nasal tip, the septum deviation correction is only partial, and this may result in bad outcomes in terms of nasal obstruction correction, besides symptom recurrence in the post-op^2^, ^5^, ^6^, ^7^, ^8^, ^9^, ^10^, ^11^, ^12^.

Those techniques which require removing the anterior portion of the cartilage with the deformity, despite having excellent results as far as nasal obstruction and nasal tip deformities go, are based on more radical and complex procedures, usually after open rhinoplasty or exo-rhinoplasties^13^, ^14^, ^15^, ^16^, ^17^, ^18^, ^19^, ^20^, ^21^.

Prospective clinical trials with strict scientific methodologies are rare insofar as nasal septum deviation correction surgeries are concerned. In a systematic metanalysis literature review, Singh et al., found 942 papers. Of these, only 13 were prospective studies of the nasal septum surgery benefits, with objective assessment methods. Acoustic rhinometry was used for the objective analysis of the results in only 2 of these studies^22^.

## OBJECTIVE

To use preliminary results to assess the efficacy of a surgical technique used to correct caudal deviations of the nasal septum.

## MATERIALS AND METHODS

This is an uncontrolled and non-randomized clinical-prospective trial, which started in June of 2007 in two university hospitals. The study was approved by the Ethics in Research Committee of the institution, under protocol # 0292/07, in of March of 2007.

Initially, we included 10 patients older than 16 years, of both genders. All of them complained of nasal obstruction without improvement with clinical treatment, associated or not with allergies and nasal cosmetic complaints, besides deviations of the septal cartilage in the areas I and II of Cottle (anterior or caudal deviations), associated or not to inferior nasal conchae hypertrophy.

We excluded from the study those patients with other rhinosinusal diseases and with a past of nasal surgeries.

The patients were submitted to otolaryngological exam, including nasal fibroscopy.

They answered a standardized questionnaire before the surgical treatment and 60 days after it. The questionnaire used was the Nasal Obstruction Septoplasty Effectiveness (NOSE) - validated by the American Academy of Otolaryngology and Head and Neck Surgery, which is specific for nasal septum deviations, with good levels of deployment, response and reading, and one which can be used as follow up in groups of patients before and after different types of clinical or surgical treatments. This is a scale with five questions about nasal symptoms to which patients assign scores varying between 0 and 4, according to symptom intensity. At the end, the total score given by the patient is multiplied by 5, and one has scores which vary between 0 - patients without symptoms, and 100 - patients with the most intense possible symptoms^23^, ^24^, ^25^, ^26^, ^27^ (Table 1).

**Table 1:** NOSE Questionnaire

	NO COMPLAINTS	MILD	MODERATE	BAD	SEVERE
1 Nasal congestion	0	1	2	3	4
2 Nasal obstruction	0	1	2	3	4
3 Problems in breathing through the nose	0	1	2	3	4
4 Sleeping problems	0	1	2	3	4
5 Difficulties in breathing through the nose during exercise or physical effort	0	1	2	3	4

Total (x5): 0-100

The patients underwent CT scan of the nasal septum in order to document the deviation and to rule out other nasal cavity and paranasal sinuses changes.

Routine preoperative and pre-anesthesia exams were carried out.

Standardized photographic documentation in six different positions was carried out before and after 60 days of the surgery in order to show a cosmetic deformity before the procedure and its improvement after the surgery.

Acoustic rhinometry was used as an objective assessment method, enabling the measurement of the minimum cross-sectional area of both nasal cavities, besides its distance in relation to the nostril opening, carried out according to the standards established in 1998.

We obtained three curves from each nostril of the patient seating down. Five minutes after applying a vasoconstriction agent we obtained three measures from each nostril. For statistical analysis purposes, we used the mean values from these measures.

In order to do an objective comparison of the postsurgical results we used the values from the *Minimal Cross Sectional Area* (MCA) and its distance in relation to the nostril opening, expressed respectively in square centimeters and centimeters^24^, ^25^, ^26^.

The patients were then submitted to surgery in the Surgical Center of two university hospitals, under general anesthesia, using the caudal or anterior septum deviation correction technique, which has been used by the authors since 2004.

All the procedures were carried out by the author of this paper and third-year residents in ENT assisted.

### Surgical technique

The technique starts by injecting local anesthetic (2% ropivacaine) and 1:100,000 epinephrine in the nasal mucosa.

Following that, we do a unilateral incision in the anterior portion of the nasal septum. The incision is preferably done on the side where the nasal septum deviation was most pronounced.

**Figure 1:**
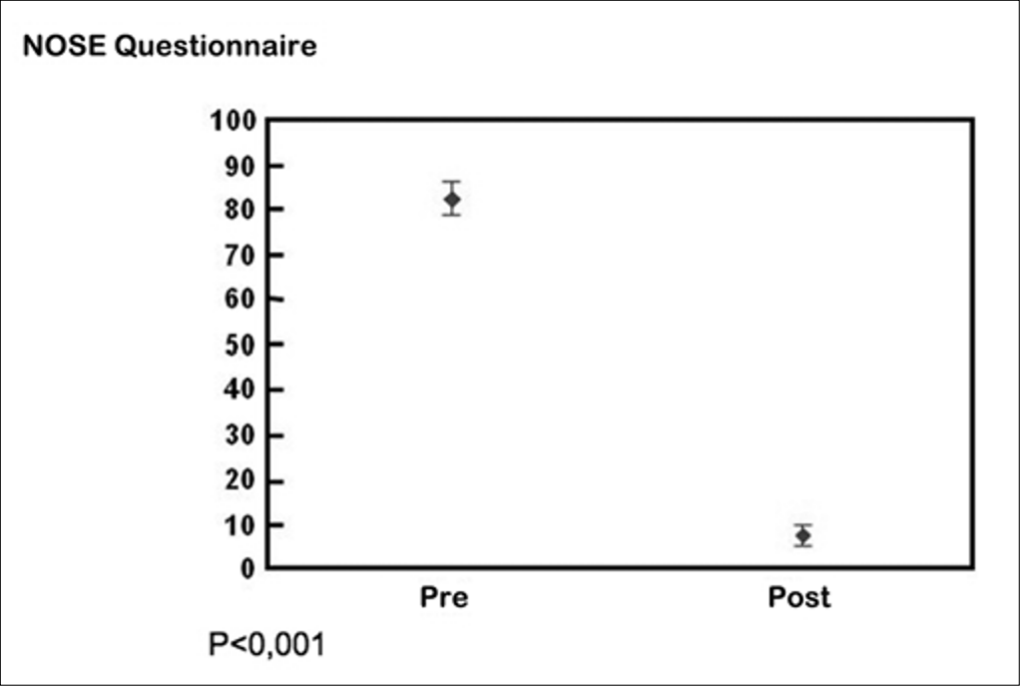
Bilateral mucoperichondrium lifting, starting at the caudal nasal septum.

We then detach the mucoperichondrium with the suction-lifter on this side, then we do it on the contralateral side (Figure 1).

**Figure 2:**
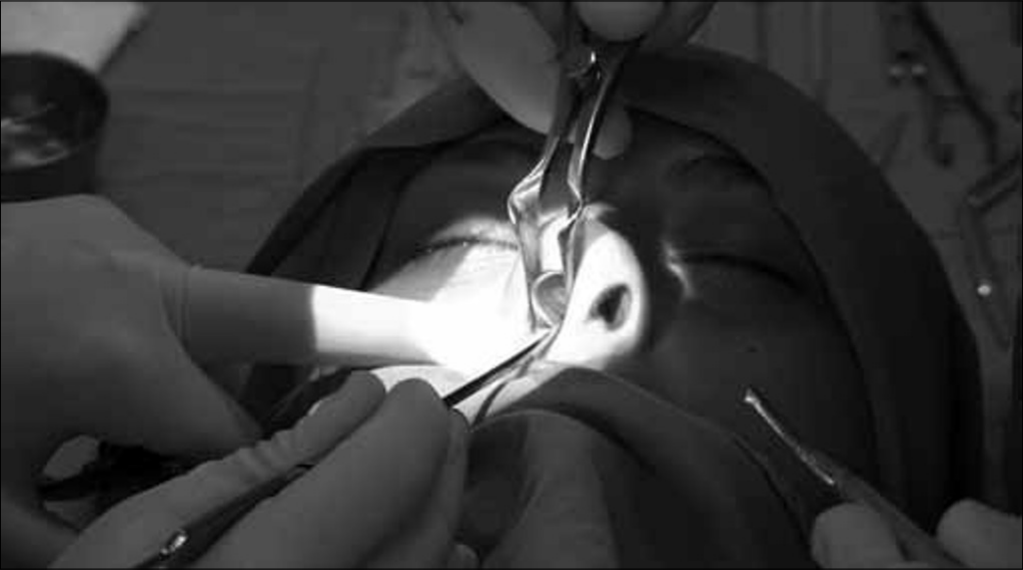
Removed the entire anterior portion with the deviation, with the posterior portion. Notice that the supra tip and nasal dorsum regions were kept intact.

After exposing the entire nasal septum cartilage, we remove the entire anterior deviated portion, and also part of the posterior cartilage, enough to make the graft which will be used to rebuild the nasal tip. The supratip and nasal dorsum portions are kept intact (Figure 2).

**Figure 3:**
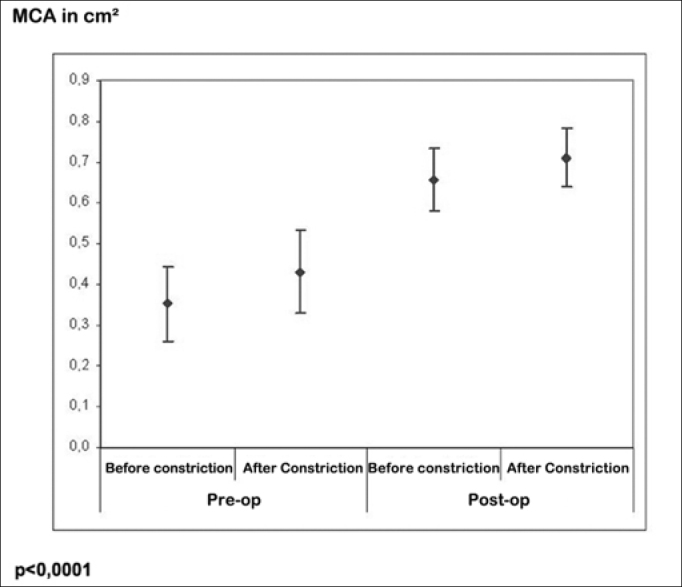
Columella strut graft making.

A strut graft is shaped from the part removed from the nasal septum, using the portion that is intact. It must be rectangular in shape, with 0.5 to 1.0 cm in width. The graft height must be assessed in each case and it must have at least the same height of the patient's nasal tip before surgery (Figure 3).

**Figure 4:**
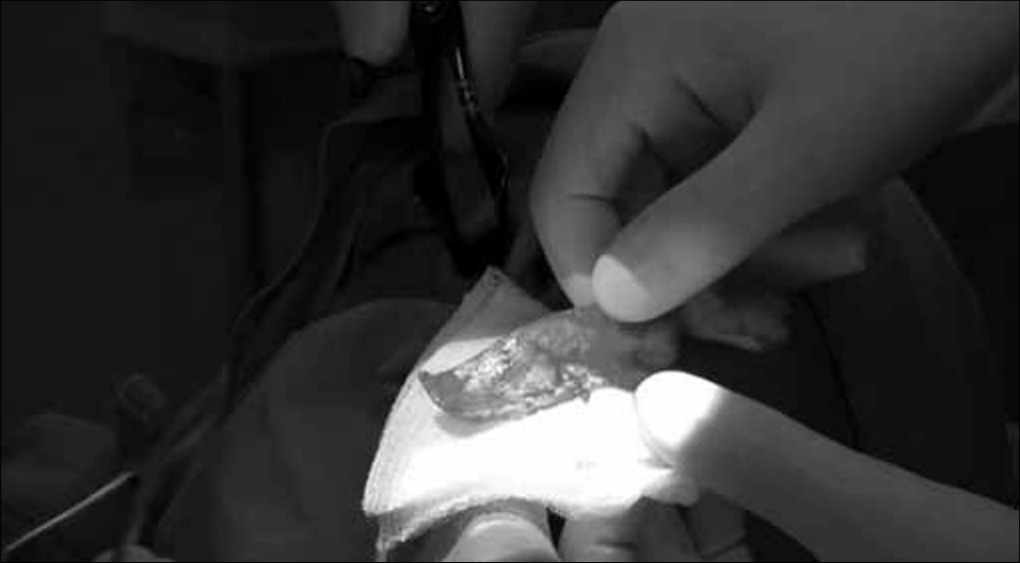
Making the tunnel between the medial crus of the alar cartilages with the angled converse scissors or curved iris scissors.

Then, from the same incision in the septal mucosa, by a retrograde way, we make a tunnel between the mucosal walls of the columella, separating the medial crus of the alar cartilages, a tunnel big enough to accommodate the graft. For that, we use a Converse or curved Iris angled scissors (Figure 4).

**Figure 5:**
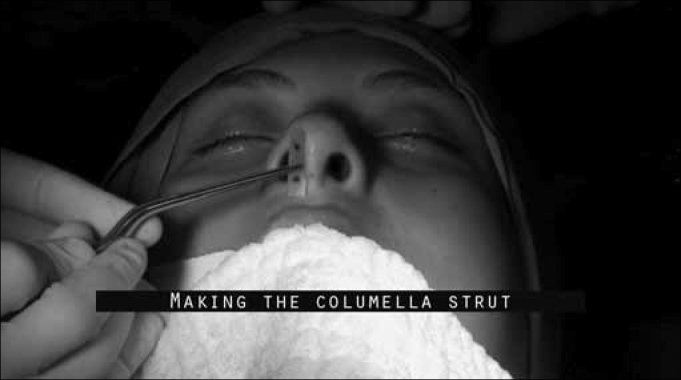
Retrograde placement of the strut. Notice the nylon stitches anchored in the graft, which help its positioning.

From the anterior portion of the columella we pass two 3-0 nylon wires, inside the tunnel, through the graft and coming back from inside the tunnel and the columella. These wires will serve to pull the graft to its correct position. It is then placed inside the tunnel between the columella mucosas in order to support the nasal tip (Figure 5).

**Figure 6:**
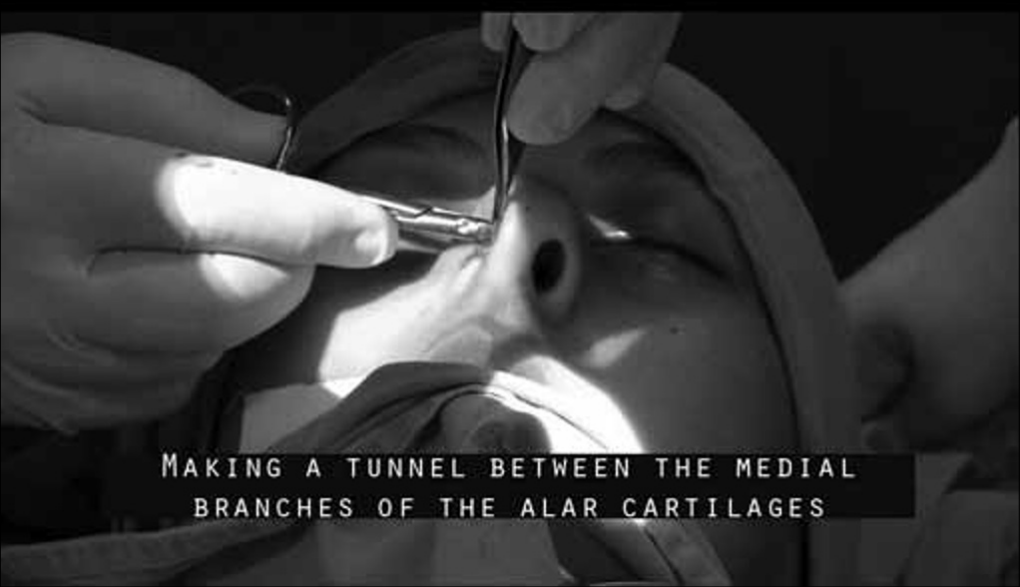
Transfixion sutures with 5-0 monocryl wire to stabilize the graft.

We then do a transfixion suture with 5-0 monocryl wire in order to stabilize the graft (Figure 6).

Transfixion sutures in the septum mucosa are done in order to have a better coaptation and to avoid hematomas.

In all the cases we did partial inferior turbinectomy, followed by minimum cauterization of the surgical beds.

We did not use splints or nasal packing.

In the statistical analysis we used the T-student test in order to assess the results from the NOSE questionnaire and the ANOVA test in order to compare the different variables from the acoustic rhinometry.

## RESULTS

A 10-patient series was prospectively followed up and submitted to surgery under the aforementioned technique. Of these, 6 (60%) were males and 4 (40%) were females.

Upon anterior rhinoscopy, 3 (30%) patients had right-side deviations and 7 (70%) had left-side deviations.

Of these, 7 cases (70%) were considered deviations causing severe obstruction (using the MCA - *Minimal Cross Sectional Area* - scale) less than 0.4 cm^2^, according to studies from Warren who used these values as minimum for maintaining nasal breathing (30).

**Graph 1:**
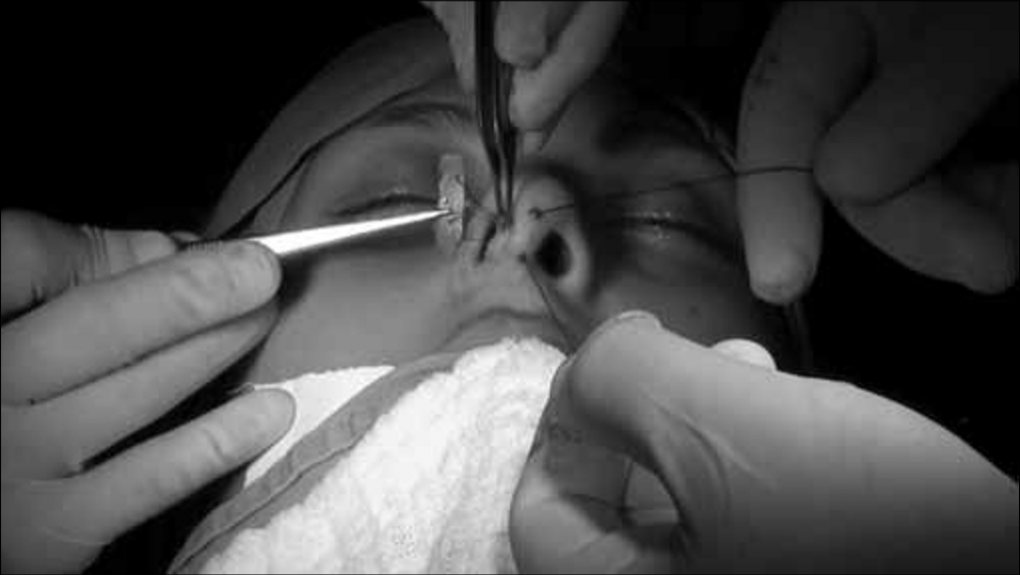
NOSE Questionnaire Results, before and after surgical treatment

Considering the results achieved based on the answers given to the standardized NOSE questionnaire, before and after surgery, we have the following results expressed on the following tables (Table 2) (Graph 1).

**Table 2:** Results from the NOSE Questionnaire

	Pre-operative	Post-operative
Mean	83.48	7.56
Standard deviation	7.23	5.91
N	10	10

*p* < 0,001

**Graph 2:**
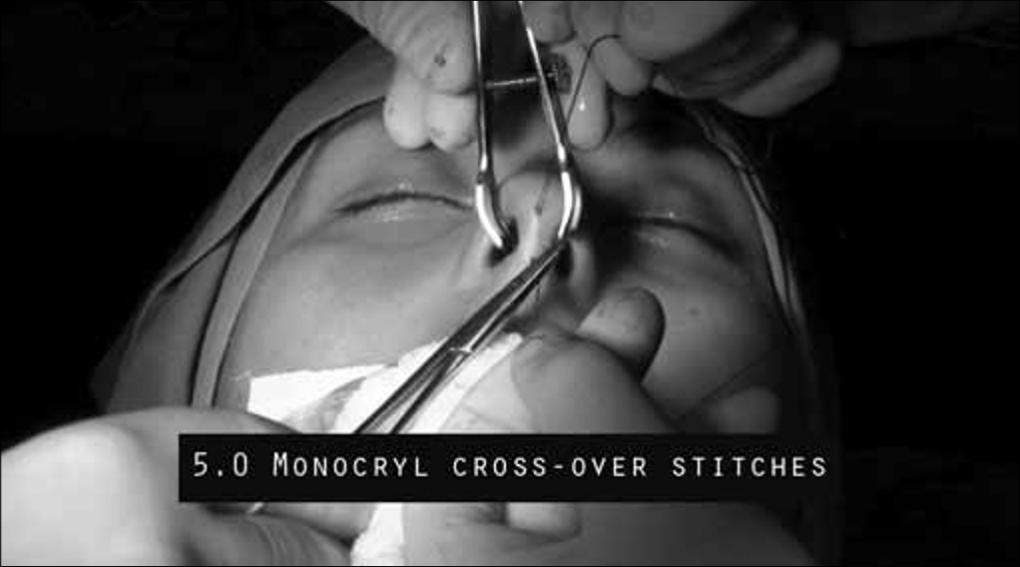
MCA (*Minimal Cross Section Area*) results, before and after surgical treatment

In order to better analyze the technique, we compared the values found in the Acoustic Rhinometry before and after the surgical correction only on the deviated side, achieving the following MCA results: (Table 3) (Graph 2).

**Table 3:** MCA results (Minimal Cross Section Area) in cm^2^

	Pre-op		Post-op	
	Before constriction	After constriction	Before constriction	After constriction
Mean	0.348	0.427	0.673	0.728
Standard deviation	0,222	0,237	0,176	0,169

N	10	10	10	10

*p*<0,0001

## DISCUSSION

The techniques most used to correct nasal septum caudal deviations have been, since their description in 1929 by Metzembaun, the so-called “swinging door” or its variations. In this type of correction, the anterior portion of the nasal septum which has the deviation is not removed, and the cartilage is mobilized by a number of incisions, or it's weakening by means of incisions on the opposite side of the deviation, followed by stabilization sutures.

The arguments against this type of technique state that by not removing the deviated portion of the cartilage, especially those located in the nasal valve region, one would not be properly correcting the deviation and the patient would continue having nasal obstruction. We must also consider that the cartilage which was not removed has a memory, in other words, it remains deviated or twisted after partial corrections. Studies from Murakami et al.^28^ showed through experiments the biomechanical particularities of the septal cartilages, and proved that partial incisions in them do not provide constant not predictable corrections, which would explain the large number of recurrence in deviations and obstructive complaints from patients submitted to these more conservative techniques, such the “ swinging door” or “morselization”.

Moreover, the conservative approaches do very little or nothing at all to change the nasal tip shape, keeping the asymmetry and the disarray caused by these deviations.

Conservative corrections of caudal deviations would then be indicated only in those mild deviations, with little obstruction complaints associated, and without asymmetries or cosmetic deformities of the nasal tip.

The other approach which can be used to correct caudal deviations are the ones which remove part of the deviated cartilage, even if it is supporting the nasal tip, and place part of the autologous cartilage in its place in order to maintain the nasal structure. This type of technique was initially described by Peer, in 1937, and the technique hereby presented is but a variation of the latter and has been in use in our service since 2003.

The critics of these techniques which remove the deviated cartilage, argue whether changes in the nasal tip structure would not be maintained even after due reconstruction. Our results, proven by post-op photographic reconstruction, show that there was no cosmetic change in relation to nasal tip support after the proposed surgical correction. Our experience with more than 6 years doing this procedure corroborates these results.

Another failure pointed out in these corrections with complete removal of the anterior septum would be that this technique is only possible through an open rhinoplasty approach, as described by some authors^19^, ^20^, ^21^. We agree that the approach through this access is really labor-intensive and, despite facilitating the exposure of these cartilages and their handling, it requires skill and experience by the surgeon in the field of rhinoplasty.

Thus, as in the studies by Grymer et al.^3^ and Dinis et al.^4^, the results from the NOSE questionnaire carried out before surgery show a large incidence of obstruction complaints from those patients with deviations in their caudal septum. The results as to the subjective improvement after surgery with the caudal septoplasty technique through the use of the same questionnaire were also statistically significant, similarly to what we found in other papers which assessed patient satisfaction after surgical correction of nasal septum deviations^15^, ^23^, ^25^, ^27^, ^29^.

Data from acoustic rhinometry also match the ones already presented in the world literature, since its development and its use to measure the results of septoplasty.

The clear increase in MCA values was statistically associated with the improvement in nasal obstruction, subjectively found through the questionnaire.

The measure of MCA values in both sides was similar to the mean values found in the general population; however, if we analyze these data separately, we notice that 14 of the 23 patients had MCA value in the obstructive side (side of the deviation) lower than 0.4 cm^2^, which classifies them as severe nasal obstruction caused by nasal septum deviation^30^.

In all the cases, after using nasal vasoconstrictor, the MCA values had a significant increase, both before and after surgical treatment.

In our results, the MCA distance values in relation to nostril opening was mildly lower than the average found in other studies, especially if we consider the control groups without nasal obstruction complaints. This is explained by the fact that all the patients in our study complained of nasal obstruction, and even for the fact that all the patients in our study had anterior nasal septum deviation.

In our bibliographic survey, of the more than 16 publications describing the techniques available to correct caudal septum deviations, most were done in a descriptive fashion, based on retrospective data and without tools for an objective checking of the results. It was only the study on the anterior septoplasty by Calderon-Cuellar et al^6^. that was carried out in a prospective fashion and used objective (rhinometry) and subjective (standardized questionnaires) methods in order to prove its efficacy.

Even considering that for caudal deviation correction techniques, and also for all types of septoplasty, there are very few prospective studies and studies with objective methods of assessment. In a metanalysis carried out in 2006, involving more than 940 papers on septoplasty, Sing. et al^22^. found only 9 using some objective methodology to prove surgical efficacy. Of these, only two had pre and postoperative data using acoustic rhinometry, which is considered the test which most depicts the nasal cavity, its narrowing and obstruction sites.

We chose to do partial inferior turbinectomy in all the patients in order to avoid a measure bias, since we could be analyzing different MCA values, which would confound the final results achieved with the surgical technique. Another attempt to minimize the anterior obstructive effect, caused by the inferior nasal conchae, was doing the measures without using vasoconstrictor agents. This way, with vasoconstriction, the mucosal hypertrophy of the nasal conchae mucosa would not be impacting MCA results. This vasoconstriction effect is clear in our results when we compare the MCA distance all the way to the nostril opening. After using nasal vasoconstriction, the distances become shorter, in other words, the MCA shifted anteriorly. This finding matches those initial studies from Grymer et al., and it was explained as an attempt to quantify the mucosal effect and that of the nasal framework on nasal obstructions^3^. In this same study, they concluded that in severely obstructive deviations of the anterior nasal septum, one must always treat the inferior nasal conchae hypertrophy.

The technique hereby described does not require specific surgeon knowledge about nasal aesthetics, it does not add any other incision or scar except for the same ones used for regular septoplasty and it does not increase the risk or likelihood of complications. The teaching of this technique to second-year residents of ENT in our service proves its applicability.

There was no cosmetic complication and no patient complained of changes, especially those associated with asymmetry or nasal tip projection. This is clear through photographic documentation of the cases studied, some with more than one year of postoperative follow up, besides the very experience of the service - where this surgery has been performed for more than 5 years, without cases of ptosis or any other nasal tip change.

## CONCLUSION

The results from this study, although still preliminary and with a small number of cases, indicate, subjectively, through patient satisfaction questionnaire, as well as objectively through acoustic rhinometry, that the technique hereby presented for the correction of nasal septum deviation seems to be efficient.
